# Anatomical repair and ligament bracing of Schenck III and IV knee joint dislocations leads to acceptable subjective and kinematic outcomes

**DOI:** 10.1007/s00167-021-06501-2

**Published:** 2021-03-10

**Authors:** Thomas Rosteius, Birger Jettkant, Valentin Rausch, Sebastian Lotzien, Matthias Königshausen, Thomas Armin Schildhauer, Dominik Seybold, Jan Geßmann

**Affiliations:** 1 Department of General and Trauma Surgery, BG University Hospital Bergmannsheil, Bürkle- de- la- Camp Platz 1, 44789 Bochum, Germany; 2 OPND Orthopädie Unfallchirurgie Praxis/Klinik Neuss, Plange Mühle 1, 40221 Düsseldorf, Germany

**Keywords:** Knee dislocation, Multiligament injuries, Anatomical repair, Gait analysis

## Abstract

**Purpose:**

The aim of this study was to analyze the outcomes of anatomical repair and ligament bracing for Schenck III and IV knee dislocation (KD).

**Methods:**

The results of 27 patients (15 and 12 cases of Schenck III and IV KD, respectively) after a mean follow-up of 18.1 ± 12.1 months (range 6–45 months) were retrospectively reviewed. Twenty-two patients suffered high-kinetic-energy accidents, whereas five patients suffered ultralow-velocity (ULV) trauma due to obesity. The outcome measures were the Lysholm score, Hospital for Special Surgery (HSS) knee score, Knee Society Score (KSS), Knee Injury and Osteoarthritis Outcome Score (KOOS) and Short Form 36 (SF-36) score. A kinematic 3D gait analysis with five walking trials was performed to compare the patients and healthy controls.

**Results:**

The mean KSS, HSS score, Lysholm score, and KOOS were 77.4 ± 14.4, 84.6 ± 11.2, 81.5 ± 10.4, and 67.3 ± 16.8, respectively. No intra- or postoperative complications occurred. The mean range of motion deficiency compared to the healthy side was 24.4 ± 18.5°. Ten patients had first-degree residual laxity of the anterior cruciate ligament; 12 and 2 patients had first- and second-degree residual laxity of the collateral ligament, respectively. Five patients underwent additional arthroscopic arthrolysis due to arthrofibrosis at an average of 6.2 ± 1.9 months (range 4–9 months) after the initial surgery. The 3D gait analysis showed no major differences in joint stability or movement between the patients and healthy controls. Only the ULV trauma patients had significantly lower outcome scores and showed larger kinematic deviations in joint movement during the gait analysis.

**Conclusion:**

Anatomical repair with ligament bracing is a suitable surgical procedure in the treatment of KD and provides evidence in clinical practice with the benefit of early, definitive repair and preservation of the native ligaments. Patients reach acceptable subjective and objective functional outcomes, including mainly normalized gait patterns during short-term follow-up, with only minor changes in kinematics and spatial–temporal characteristics. Obese patients who suffered ULV trauma showed significantly inferior outcomes with larger deviations in joint kinematics.

**Level of evidence:**

Level III.

**Supplementary Information:**

The online version contains supplementary material available at 10.1007/s00167-021-06501-2.

## Introduction

Knee dislocation (KD) with multiligament lesions is one of the most severe injuries of the knee joint, not least due to the high rate of accompanying injuries, such as peroneal nerve, vascular, chondral and meniscal lesions, injury to the posterolateral capsule and/or popliteus complex or disruption of the m. biceps femoris [3, 8, 10, 16, 17, 25, 37, 40, 51] and complications like the need for blood transfusion or pulmonary embolism [27]. The occurrence of KD has been reported to account for between 0.02% and 0.1% of all musculoskeletal injuries [40]. The injury mechanism of KD is quite inconsistent and is mainly divided into high-and low kinetic-energy trauma [11], for example, sports or work accidents, and ultralow-velocity (ULV) trauma (activities of daily living) with high energy related to obesity [16]. Since nonsurgical therapy in the case of acute KD yields unsatisfactory results [14, 29, 35, 38], various surgical procedures have been developed. These strategies range from early to late surgery [18] and repair to reconstruction and one- to two-stage procedures, with comparably satisfactory results [1, 4, 9, 15, 19, 24, 30, 34, 43, 54], even if most authors recommend a staged procedure for the repair of peripheral structures and reconstruction of cruciate ligaments [5, 14, 16, 22]. Furthermore, both open and arthroscopic treatment strategies in the early and late stages have been described as practicable [12, 20, 46]. However, due to the inhomogeneity of the patient characteristics, small case numbers and different associated injuries, no standards of treatment currently exist [32]. Therefore, Heitmann et al. presented the first results of a multicenter study of anatomical repair and ligament bracing as a new treatment option for acute KD [16]. The obtained results and revision rate show that early primary suture repair is a promising option [16], even if ULV KD seems to be a particular problem, with an increasing incidence over recent years, higher rates of concomitant injuries and complications and poorer outcomes [16, 23, 39, 50]. However, only a few studies are available regarding the outcome of anatomical repair with suture augmentation after acute KD. Furthermore, to the best of our knowledge, 3D gait analysis following the surgical repair of acute KD has not yet been performed. Gait analysis allows objective kinematic measurements to be obtained and has been used for the evaluation of different knee surgeries, including total knee arthroplasty [2, 53] and anterior cruciate ligament (ACL) reconstruction [44]. Therefore, the aim of this study was to analyze the subjective and functional outcomes of anatomical repair and ligament bracing for Schenck III and IV acute KD to evaluate the clinical suitability and thereby improve the management of this severe injury. It was hypothesized that anatomical repair and ligament bracing yield good functional results, including widely restored physiological gait kinematics.

## Materials and methods

The study was reviewed and approved by the local ethics committee of the medical faculty of Ruhr University Bochum, Germany (registered number: 18-6508_1-BR). All procedures were performed in accordance with the ethical standards of the institutional research committee and with the 1964 Declaration of Helsinki and its later amendments.

Patients with acute KD undergoing anatomical repair and bracing of the ruptured ligaments between 01/2015 and 01/2019 were retrospectively reviewed. KD was categorized according to the classification reported by Schenck et al. [42], which subdivides the severity of the injury based on the ruptured ligaments. Only patients presenting with clinical and radiological evidence of type III or IV KD were included in this study. The other inclusion criteria were clinical examination and gait analysis data with a minimum follow-up of 6 months. Patients with polytrauma, with type I or II KD, treated with a hybrid technique (ligament reconstruction and/or suture or two-stage procedures) or with incomplete data were excluded from further analysis. In total, 27 of 33 (81.8%) patients (15 and 12 cases of Schenck III and IV KD, respectively) with a mean follow-up of 18.1 ± 12.1 months (range 6–45 months) fulfilled the inclusion criteria and were considered for further analysis. Twenty-two of these patients suffered high- or low-velocity accidents, whereas five patients underwent ULV trauma due to obesity (mean BMI, 44.1 ± 11.6 kg/m^2^). Table 1 shows data regarding the demographics, allocation and concomitant injuries in the study group.

**Table 1:** Study group

Study group	
Age (years)	38.3 ± 14.4 (range 15–61)
Sex	
Male	18 (66.7%)
Female	9 (33.3%)
ASA-score	
I	15 (55.6%)
II	10 (37.0%)
III	2 (7.4%)
Comorbidities	
Smokers	9 (33.3%)
Arterial hypertension	3 (11.1%)
Diabetes mellitus	1 (3.7%)
Asthma	1 (3.7%)
Epilepsy	1 (3.7%)
BMI (kg/m^2^)	29.0 ± 9.3 (range 15.6–60.1)
Type of injury	
KD 3 medial	6 (22.2%)
KD 3 lateral	9 (33.3%)
KD 4	12 (44.4%)
Concomitant injuries	
Disruption of posterolateral capsule and/or popliteus complex	15 (55.6%)
Peroneal nerve lesion	5 (18.5%)
Meniscal lesion	8 (29.6%)
Chondral lesion	1 (3.7%)
Disruption of m. biceps femoris	3 (11.1%)
Disruption of m. vastus medialis	1 (3.7%)
Fracture of fibula head and/or tibial plateau and/or distal femur	2 (7.4%)

### Surgical management and postoperative procedure

Anatomical repair with ligament bracing was performed based on plain radiographs, MRI scans and intraoperative findings in combination with a physical examination of ligamentous instability. Prior to open reconstruction, diagnostic arthroscopy was performed to identify possible meniscal tears or chondral lesions. An augmented primary suture repair of all torn ligaments was then performed using the surgical technique previously described by Heitmann et al. [16]. In brief, an anteromedial parapatellar arthrotomy was used to address type III medial KD. In the case of type III lateral KD, injury of the posterolateral corner or type IV KD, an additional lateral incision was performed. ACL and posterior cruciate ligament (PCL) tunnels were drilled in standard positions with the assistance of arthroscopic ACL and PCL drill guides. The ligament stumps were reinforced with FiberWire 2 (Arthrex, Naples, USA), and FiberWire 5 was used in placing augmentation sutures. After extracortical diversion through the drill tunnels, the sutures were knotted using metal suture buttons (Arthrex, Naples, USA). First, the augmentation sutures were knotted, followed by tension-free knotting of the pull-out sutures of the ligaments [16]. After reconstruction of the cruciate ligaments, all torn collateral ligaments were repaired using transosseous pull-out sutures that were fixed extracortically with suture buttons. In cases of posterolateral corner injuries with avulsion of the popliteus tendon, the tendon was also fixed with a transosseous pull-out suture. In all cases of a meniscal tear, open repair was performed; in one case, debridement and microfracture were performed for a grade 4 chondral lesion.

Physical therapy started 48 h after the operation with passive motion of the joint in the prone position with limited range of motion (ex./flex. 0°/0°/90°). In some cases, peripheral nerve block anesthesia was applied. Patients had a limited weight-bearing of 20 kg and limited range of motion (ex./flex. 0°/0°/90°) for 6 weeks. Flexible stabilization braces were recommended for 12 weeks (e.g., a DonJoy Armor^®^ brace).

### Follow-up examination

The patient assessment and clinical examination were scheduled at a minimum of six months after the primary surgery. Subjective and functional outcomes after ligament bracing were determined using the Lysholm score [47], Knee Injury and Osteoarthritis Outcome Score (KOOS) [41], Hospital for Special Surgery (HSS) knee score [21], Knee Society score (KSS) [33] and Short Form 36 (SF-36) score [49]. Time to return to work and return to sport with regard to cycling, swimming and walking were recorded. Additionally, to quantify the kinematics (e.g., side-by-side differences, stability and movement) of the joints of the lower extremities, a 3D gait analysis (3D myoMOTION, Noraxon, Scottsdale, USA) using inertial sensor technology was performed.

### Gait analysis

The 3D biomechanical gait analysis was conducted while the participant performed five trials of level walking over 10 m at a self-selected pace. Therefore, the 3D myoMOTION system (Noraxon, Scottsdale, AZ, USA) was used. The system consists of seven sensors and is based on inertial sensor technology. The sensors have a maximum sampling rate of 200 Hz. The accuracy of anatomical angles in the static setup is ± 1°, and that in the dynamic setup is ± 2°. The accuracy of the orientation angles for the pitch and heading is 0.25° and 1.25°, respectively. The sensors were mounted in the designated positions at the pelvis, thighs, shanks and feet. Based on a so-called fusion algorithm, the information from a 3D accelerometer, gyroscope and magnetometer is used to measure the anatomical angles of the pelvis, hips, knees and ankles. MR 3.14 software (Noraxon, Scottsdale, AZ, USA) was used to calculate the values of the joint angles from the inertial sensor data. The sensors were calibrated before each walking trial separately. The validity and reliability of wearable inertial measurement units have received particular attention in the area of analyzing spatiotemporal characteristics and gait parameters [26]. The mean and peak angles of the joints during the stance and swing phases of walking, the walking speed and the time of double limb support were analyzed. Gait data were evaluated side by side. The kinematic curves normalized to a gait cycle in the patients were compared to those of an age-, sex- and BMI-matched control group of 20 healthy participants without injuries or a history of surgery in the lower extremities (excluding morbidly obese patients).

### Statistical analysis

Descriptive data are described by the mean, standard deviation, minimum and maximum. After the normality of the data was tested using the Shapiro–Wilk test, normally distributed variables were assessed using the two-tailed *t*-test, a parametric test. Nonnormally distributed variables were analyzed with the Wilcoxon/Mann–Whitney test. Nominally scaled variables were compared using cross tables and Fischer’s exact test. To assess the level of similarity between the different kinematic curves normalized to the gait cycle, cross-correlation analysis was performed. The sample size calculation using G*Power (version 3.1.9.6 for Mac, University of Dusseldorf, Germany) resulted in a minimum sample size of 19 persons for both the study and control group. The gait data were processed in MATLAB, MathWorks, Inc. The level of statistical significance was set to α = 0.05.

## Results

The subjective and functional outcome scores are shown in Tables 2 and 3. Patients with ultra-low velocity (ULV) trauma had a significantly lower KSS, HSS knee score and SF-36 Physical Functioning, Energy/Fatigue, Emotional Well-Being and Social Functioning score (Table 4). All patients reached full weight-bearing without the use of canes, crutches or external braces.

**Table 2:** Overall outcome scores in the study group

	KSS	KSS functional	HSS Knee Score	Lysholm Score	KOOS
Mean	77.4	80.2	84.6	81.5	67.3
Standard deviation	14.4	20.3	11.2	10.4	16.8
Minimum	41	20	55	67	38.7
Maximum	100	100	100	96	95.2

*KSS* Knee Society Score, *KOOS* Knee Injury and Osteoarthritis Outcome Score

**Table 3:** SF-36 scores

SF 36%	Physical functioning	Role limitations (physical health)	Role limitations (emotional problems)	Energy/fatigue	Emotional well-being	Social functioning	Pain	General health	Health change
Mean	67.3	52.8	67.9	60	74.1	83.4	71.5	70.9	67.6
SD	21.5	42.1	41.1	20.6	20.3	24.6	26.4	16.0	26.2
Minimum	20	0	0	15	16	0	22.5	45	0
Maximum	100	100	100	95	96	100	100	95	100

**Table 4:** Significant differences in outcome scores between ULV and HV + LV trauma patients

	HV + LV	ULV	
	Mean ± SD	Mean ± SD	*P* value
Knee Society Score	80.7 ± 13.7	62.6 ± 8.9	0.010
Knee Society Score funct	86.6 ± 13.4	52.0 ± 24.9	0.000
HSS Knee Score	88.0 ± 8.8	69.4 ± 8.8	0.000
SF 36 physical functioning %	73.3 ± 17.6	41.0 ± 20.7	0.001
SF 36 energy/fatigue %	64.3 ± 18.4	41.0 ± 23.0	0.022
SF 36 emotional well-being %	79.7 ± 13.9	49.6 ± 29.2	0.002
SF 36 Social functioning %	88.2 ± 22.0	62.5 ± 29.3	0.035

*HV* high-velocity trauma, *LV* low-velocity trauma, *ULV* ultra-low velocity trauma

In total, 24 patients returned to work at an average of 7.8 ± 4.0 months (range 1–18 months), and 21 patients returned to sports (cycling, walking, swimming) at an average of 8.7 ± 4.4 months (range, 4–24 months) after the operation. However, the activity level of sports was lower after the accident in 17 patients. The mean range of motion deficiency compared to the healthy side was 24 ± 19°, but only five patients had an extension deficit of 5°. The thigh and shank circumference as a measure of muscle atrophy was reduced on the operative side compared to the healthy side by an average of 1.9 ± 1.5 cm and 1.1 ± 1.0 cm, respectively. Table 5 demonstrates the residual laxity after ligament bracing of the knee joint. No intra- or postoperative complications occurred during the study period. In five patients, additional arthroscopic arthrolysis due to arthrofibrosis was performed an average of 6.2 ± 1.9 months (range 4–9 months) after the initial surgical treatment.

**Table 5:** Remaining postoperative functional deficits and laxity of the ligaments

Residual deficit	
ROM deficit	24 ± 19° (range 0–75°)
ACL laxity	10 patients (1°)
PCL laxity	0 Patients
Collateral laxity	
Lateral Medial Medial + lateral	9 Patients (8 patients 1°, one patient 2°) 3 Patients (1°) 1 Patient (2°)

### Gait analysis

**Fig. 1:**
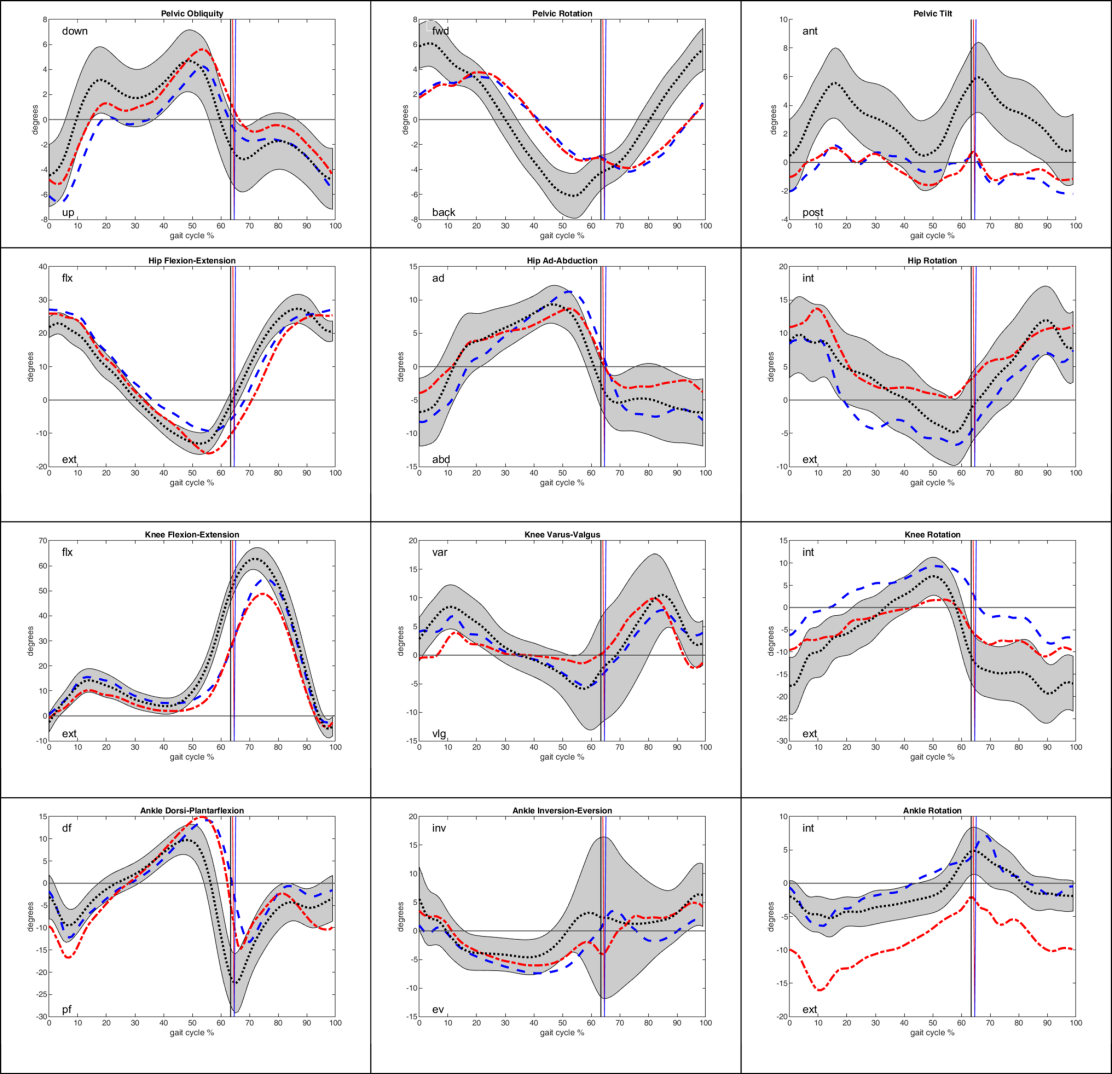
Kinematic curves for peak joint angles on the healthy side (blue) and injured side (red) of ULV trauma patients in contrast to the mean curve in the healthy control group (black). The gray area demonstrates the range of the standard deviation in the control group. The vertical lines mark the transition from stance to swing phase

**Fig. 2:**
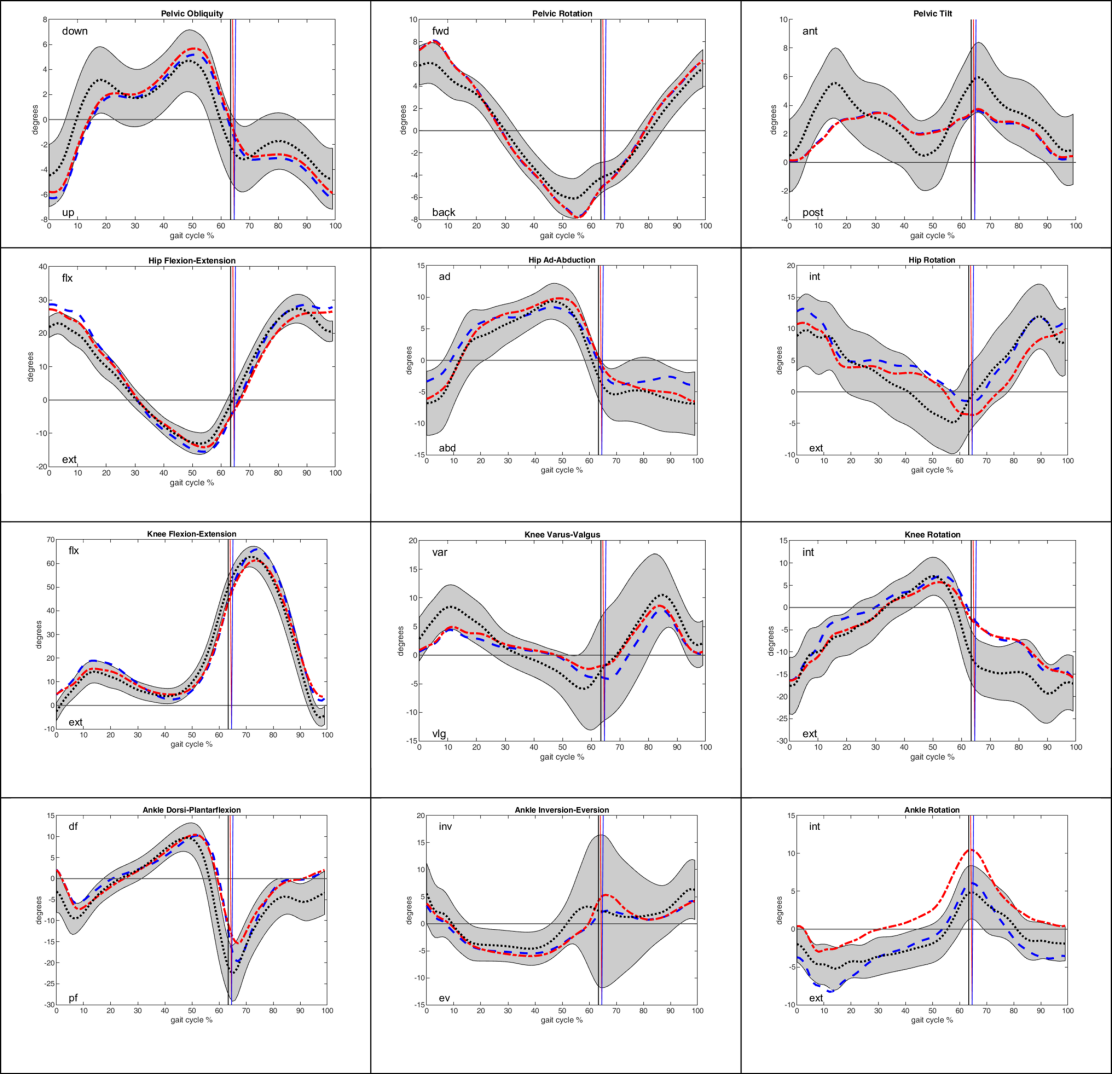
Kinematic curves for peak joint angles on the healthy side (blue) and injured side (red) in the study group without ULV trauma patients in contrast to the mean curve in the healthy control group (black). The gray area demonstrates the range of the standard deviation in the control group. The vertical lines mark the transition from stance to swing phase

Spatial–temporal characteristics and differences between the control and patient groups determined by gait analysis are shown in Table 6. In particular, the patients had a significantly longer stance phase and shorter swing phase than did the controls, although the velocity of the patients was significantly slower. Kinematic curves of the different patient groups in relation to the control group are demonstrated in Figs. 1 and 2. The kinematic curves of the patient group without ULV trauma deviated only slightly from the mean kinematic curve and standard deviation of the control group (Fig. 2). The morbidity obese patients with ULV trauma showed larger deviations in gait kinematics than the controls (Fig. 1). The additionally calculated cross-correlation of the gait curves demonstrated a high similarity of the kinematic curves of the patients and controls.

**Table 6:** Spatial–temporal characteristics

	ULV (*n* = 5)	HV + LV (*n* = 22)	Control (*n* = 20)
Stance %	67.6 ± 2.3 H* (*p* < 0.001) 66.4 ± 2.6 I* (*p* < 0.001)	64.3 ± 1.7 H* (*p* < 0.001) 63.5 ± 1.9 I (*p* = 0.246)	63.3 ± 1.4
Pre-swing %	17.2 ± 3.5 H* (*p* < 0.001) 16.8 ± 1.4 I* (*p* < 0.001)	14.6 ± 2.6 H* (*p* < 0.001) 13.3 ± 2.4 I (*p* = 0.794)	13.2 ± 1.5
Swing %	32.4 ± 2.3 H* (*p* < 0.001) 33.6 ± 2.6 I* (p < 0.001)	35.7 ± 1.7 H* (*p* < 0.001) 36.5 ± 1.9 I (p = 0.246)	36.7 ± 1.4
Double Stance %	33.9 ± 4.8* (*p* < 0.001)	27.7 ± 3.4* (*p* = 0.002)	26.3 ± 2.7
Step length cm	53.5 ± 7.3 H* (p < 0.001) 54.7 ± 9.7 I* (*p* < 0.001)	65.6 ± 8.4 H* (p = 0.009) 65.0 ± 8.8 I* (*p* = 0.002)	68.2 ± 7.6
Step time ms	611.7 ± 141.1 H* (p < 0.001) 616.8 ± 100.0 I* (*p* < 0.001)	574.2 ± 56.0 H* (p < 0.001) 568.6 ± 51.4 I* (*p* < 0.001)	539.2 ± 46.7
Velocity km/h	3.3 ± 0.8* (*p* < 0.001)	4.2 ± 0.7* (*p* < 0.001)	4.6 ± 0.6

^*^Indicates significant difference (*p* < 0.02) to control group

*H* healthy side, *I* injured side

## Discussion

The most important finding of the present study was the demonstration of the adequate suitability of anatomical repair and ligament bracing as a rarely described surgical procedure for treating high-grade KD with a low complication rate. Considering the severity of this injury, our study demonstrates satisfactory subjective and functional outcomes and largely physiological gait after the operative treatment of high-grade KD using the method of anatomical repair and ligament bracing published by Heitmann et al. [17] and Frosch et al. [14]. Most of the patients reported acceptable to good knee function, with sufficient stability and low pain. Furthermore, our short-term results show low complication and revision rates, additionally supporting anatomical repair and ligament bracing as a procedure for the successful treatment of these serious injuries. The overall health status (SF-36 score) was also mostly characterized as good. The mean postoperative stiffness of the knee with flexion deficits, muscle atrophy of the thigh and shank muscles and minor changes in gait were restrictive; however, they were considered acceptable considering the severity of the injury and current literature [1]. Nevertheless, these limitations resulted in an extended rehabilitation time and time to return to sports and work in most of the patients, who did not reach their preinjury activity level during the follow-up period. Patients with ULV trauma were particularly affected by poorer outcomes (Tables 4, 6).

These results are in agreement with the results reported by Heitmann et al. [16], who demonstrated the first results of anatomical repair and ligament bracing for KD in their prospective, multicenter study. Sixty-nine cases of KD (Schenck III and IV) were evaluated. The average International Knee Documentation Committee (IKDC) score was 75.5 ± 14.5, the average Lysholm score was 81.0 ± 15.5, and the median loss of activity according to the Tegner score was 1 (range 0–3) point; these outcomes are nearly the same as our results (Tables 2, 3). Stress radiographs showed a mean side-to-side difference of 3.2 ± 1.3 mm for the ACL and 2.9 ± 2.1 mm for the PCL. The surgical revision rate (early and late) was 17.4%. In the later stage, four patients with knee stiffness and six patients with symptomatic knee instability needed reoperation. The rate of arthrofibrosis requiring surgical intervention was 23.2% [16] (vs. 18.5% in our study). Indeed, the patients with ULV trauma, in accordance with our results, had significantly inferior outcome scores [16], probably due to the high overweight of these patients.

Moreover, as an additional outcome measure, a 3D biomechanical gait analysis was performed to analyze the kinematics of the lower extremity joints during full weight-bearing while performing a complex movement sequence. To date, no data regarding gait in KD have been reported in the literature. This gait analysis showed a largely physiological gait cycle in the short- to midterm follow-up period, with no major differences in joint kinematics between the injured and healthy sides or between the patients and healthy controls (Fig. 2). However, the spatiotemporal characteristics showed significant differences between the patients and controls, and the changes in gait speed, smaller step length and longer stance phase were certainly related to the injury (Table 6). Taking a differentiated view of the knee kinematics, slightly lower knee varus/valgus and lower external rotation while transitioning from the stance to the swing phase of the gait cycle were observed (Fig. 2). These observations are comparable to known gait data from patients treated with ACL reconstruction, which also describe changes in the frontal- and sagittal-plane walking kinematics of the knee in the early- to midterm follow-up period [44]. Looking at the subgroup of patients with ULV trauma, in accordance with the subjective and clinical results, the larger deviations in joint movement during the gait cycle are striking. For example, reduced knee rotation and knee flexion indicated increased stiffness of the operated knee joint (Fig. 1), which was in accordance with the results of the clinical examination. When considering the gait analysis, it must be mentioned restrictively that obesity has an influence on joint movement during gait [28]. Therefore, the deviations in the gait of the patients with ULV trauma could partly be due to the high overweight of these patients and the low number of patients investigated.

Considering the major advantages of this surgical procedure for anatomical repair and ligament bracing, early, timely, definitive surgical treatment after trauma [18] (in contrast to late reconstruction with a longer period of limited movement and disease progression for the patient) and preservation of the native ligaments with the possibility of subsequent reconstruction in case of remaining laxity [36, 52] must be emphasized. However, two difficulties in our patient cohort were also noticed: (1) residual laxity, especially regarding the ACL and collateral ligaments; and (2) postoperative stiffness due to arthrofibrosis requiring arthroscopic arthrolysis during the follow-up period. In that regard, whether ligament reconstruction or repair provides greater stability, reduces arthrofibrosis and consequently improves the outcomes remains a matter of debate [7, 14, 32, 48]. Regarding the residual laxity of the ACL, Heitmann et al. mentioned a hybrid technique involving ACL reconstruction and bracing of the other ruptured ligaments as a promising option [16]. Focusing on postoperative arthrofibrosis, it is unclear whether early surgical repair via arthrotomy leads to increased postoperative stiffness, whereas it is known that early reconstruction leads to a higher rate of postoperative stiffness [16]. In conclusion, it must be stated that comparative data regarding complications and outcomes between surgical repair and reconstruction after KD are missing, which is why such studies would be desirable [6, 14].

Despite trying to ensure reliability, there are certain limitations to our study. An inhomogeneous follow-up period among the patients is based on the retrospective study design. Furthermore, only short-term results with a mean follow-up of 18.1 ± 12.1 months are provided by the study. Gait analysis using inertial sensors does not provide any information about kinetics, which prevents a deeper understanding of rehabilitation mechanisms. Moreover, the small study group with varied results and distinctly pronounced differences in the gait pattern between the individual subgroups (e.g., patients with ULV trauma) led to large standard deviations, which might have led to underpowered statistical results. However, the small group size is due to the rarity of the injury and the single-center study design. Therefore, because of the high relevance of this topic, larger, longer-term investigations of this surgical procedure are required, especially since nearly about 25% of patients with KD suffer from osteoarthritis in the long-term [13, 31, 45]. Despite these limitations, anatomical repair and ligament bracing can be evaluated as a promising option for the treatment of KD based on the results of our study. This approach offers orthopedic surgeons a working treatment concept in daily clinical practice, with acceptable short- term objective outcomes, low complication rates and the advantages of early, definitive repair and preservation of the native ligaments, since the treatment of KD is still debated in the literature [32]. Moreover, in the day-by day work, the results of our study help inform patients, who suffered from KD, about first clinical results and benefits of ligament bracing.

## Conclusion

The treatment of Schenck III and IV KD by anatomical repair and ligament bracing leads to acceptable subjective and functional outcomes. Furthermore, patients who suffered from high- or low-velocity accidents showed a widely restored physiological gait pattern in the short-term follow-up. Obese patients who suffered ULV trauma showed significantly inferior outcomes and larger deviations in joint kinematics.

## Supplementary Information

Below is the link to the electronic supplementary material.

Supplementary file1 (DOCX 13 KB)

Supplementary file2 (DOCX 13 KB)

## Abbreviations

KD: Knee dislocation
ULV: Ultralow-velocity
KOOS: Knee Injury and Osteoarthritis Outcome Score
HSS: Hospital for Special Surgery
KSS: Knee Society Score
SF 36: Short Form 36
IKDC: International Knee Documentation Committee
