# Supplementary material for: Anatomical repair and ligament bracing of Schenck III and IV knee joint dislocations leads to acceptable subjective and kinematic outcomes
Source: Knee Surg Sports Traumatol Arthrosc. 2021 Mar 10;29(12):4188–97. doi: 10.1007/s00167-021-06501-2 (PMC8595154; doi:10.1007/s00167-021-06501-2)
Supplement: Supplementary file 1 — Supplementary file1 (DOCX 13 KB) [file 167_2021_6501_MOESM1_ESM.docx]

**Table 7:** Analysis of the in-range percentage of gait cycle curves compared with the standard deviation of the control group

|  | **ULV (n=5)** | | **HV+ LV (n=22)** | |
| --- | --- | --- | --- | --- |
| **In range (mean ± SD)**  **Control group** | **Healthy side**  % gait cycle | **Injured side**  % gait cycle | **Healthy side**  % gait cycle | **Injured side**  % gait cycle |
| **Pelvic Obliquity** | 81 | 89 | 93 | 100 |
| **Pelvic Rotation** | 23 | 23 | 91 | 89 |
| **Pelvic Tilt** | 26 | 21 | 90 | 90 |
| **Hip Flexion- Extension** | 18 | 56 | 68 | 73 |
| **Hip Ad- Abduction** | 86 | 94 | 100 | 100 |
| **Hip Rotation** | 86 | 95 | 88 | 100 |
| **Knee Flexion- Extension** | 75 | 54 | 57 | 72 |
| **Knee Varus- Valgus** | 100 | 72 | 87 | 94 |
| **Knee Rotation** | 20 | 58 | 80 | 81 |
| **Ankle Dorsi- Plantarflexion** | 83 | 54 | 68 | 71 |
| **Ankle Inversion- Eversion** | 87 | 97 | 100 | 100 |
| **Ankle Rotation** | 87 | 0 | 92 | 24 |
