# Supplementary material for: Anatomical repair and ligament bracing of Schenck III and IV knee joint dislocations leads to acceptable subjective and kinematic outcomes
Source: Knee Surg Sports Traumatol Arthrosc. 2021 Mar 10;29(12):4188–97. doi: 10.1007/s00167-021-06501-2 (PMC8595154; doi:10.1007/s00167-021-06501-2)
Supplement: Supplementary file 2 — Supplementary file2 (DOCX 13 KB) [file 167_2021_6501_MOESM2_ESM.docx]

**Table 8:** Cross-correlation of gait cycle curves between the patients and control group

|  | **ULV (n=5)** | | **HV+ LV (n=22)** | |
| --- | --- | --- | --- | --- |
| **Cross Correlation with Control group** | **Healthy side** | **Injured side** | **Healthy side** | **Injured side** |
| **Pelvic Obliquity** | 0.79 | 0.82 | 0.94 | 0.94 |
| **Pelvic Rotation** | 0.66 | 0.68 | 1.00 | 1.00 |
| **Pelvic Tilt** | 0.48 | 0.68 | 0.63 | 0.64 |
| **Hip Flexion- Extension** | 0.95 | 0.93 | 0.99 | 0.98 |
| **Hip Ad- Abduction** | 0.93 | 0.97 | 0.99 | 0.99 |
| **Hip Rotation** | 0.93 | 0.91 | 0.88 | 0.80 |
| **Knee Flexion- Extension** | 0.95 | 0.97 | 0.98 | 0.99 |
| **Knee Varus- Valgus** | 0.98 | 0.68 | 0.90 | 0.93 |
| **Knee Rotation** | 0.95 | 0.98 | 0.94 | 0.91 |
| **Ankle Dorsi- Plantarflexion** | 0.64 | 0.71 | 0.92 | 0.93 |
| **Ankle Inversion- Eversion** | 0.82 | 0.81 | 0.94 | 0.90 |
| **Ankle Rotation** | 0.90 | 0.91 | 0.95 | 0.97 |
